# Gene regulatory response to hyposalinity in the brown seaweed *Fucus vesiculosus*

**DOI:** 10.1186/s12864-020-6470-y

**Published:** 2020-01-13

**Authors:** Luca Rugiu, Marina Panova, Ricardo Tomás Pereyra, Veijo Jormalainen

**Affiliations:** 1 Department of Marine Sciences –Tjärnö, University of Gothenburg, SE 452 96 Strömstad, Sweden; 2 Department of Biology, University of Turku, FIN-20014 Turku, Finland

**Keywords:** *Fucus*, Hyposalinity, Climate change, Transcriptomic, Genetic variation

## Abstract

**Background:**

Rockweeds are among the most important foundation species of temperate rocky littoral shores. In the Baltic Sea, the rockweed *Fucus vesiculosus* is distributed along a decreasing salinity gradient from the North Atlantic entrance to the low-salinity regions in the north-eastern margins, thus, demonstrating a remarkable tolerance to hyposalinity. The underlying mechanisms for this tolerance are still poorly understood. Here, we exposed *F. vesiculosus* from two range-margin populations to the hyposaline (2.5 PSU - practical salinity unit) conditions that are projected to occur in the region by the end of this century as a result of climate change. We used transcriptome analysis (RNA-seq) to determine the gene expression patterns associated with hyposalinity acclimation, and examined the variation in these patterns between the sampled populations.

**Results:**

Hyposalinity induced different responses in the two populations: in one, only 26 genes were differentially expressed between salinity treatments, while the other population demonstrated up- or downregulation in 3072 genes. In the latter population, the projected future hyposalinity induced an acute response in terms of antioxidant production. Genes associated with membrane composition and structure were also heavily involved, with the upregulation of fatty acid and actin production, and the downregulation of ion channels and alginate pathways. Changes in gene expression patterns clearly indicated an inhibition of the photosynthetic machinery, with a consequent downregulation of carbohydrate production. Simultaneously, energy consumption increased, as revealed by the upregulation of genes associated with respiration and ATP synthesis. Overall, the genes that demonstrated the largest increase in expression were ribosomal proteins involved in translation pathways. The fixation rate of SNP:s was higher within genes responding to hyposalinity than elsewhere in the transcriptome.

**Conclusions:**

The high fixation rate in the genes coding for salinity acclimation mechanisms implies strong selection for them. The among-population differentiation that we observed in the transcriptomic response to hyposalinity stress suggests that populations of *F. vesiculosus* may differ in their tolerance to future desalination, possibly as a result of local adaptation to salinity conditions within the Baltic Sea. These results emphasise the importance of considering interspecific genetic variation when evaluating the consequences of environmental change.

## Background

Foundation species influence the structure and function of the ecosystems in which they live by providing physical habitat and resources for associated communities [1]. In temperate rocky shores, brown rockweeds are a foundation species for littoral communities and contribute to ecosystem function through biomass accumulation, the transfer of energy and matter to higher trophic levels, and by controlling environmental conditions such as hydrodynamic forces and sedimentation [2]. Although rockweeds are adapted to life in the highly variable littoral environment, their reproduction, growth, and survival are vulnerable to variability in environmental factors such as temperature, salinity, eutrophication, and pH [3–6]. Climate change is expected to modify all these factors, with the extent of the perturbation varying from one region to another. In general, our current knowledge of the tolerance of marine macroalgae to future climate-change conditions is limited to a few factors, mainly temperature and acidification [7, 8], but also salinity [9–11]. In the Baltic Sea, a major change induced by climate change will be desalination [12], which will shift the surface water salinity gradient southward and challenge the persistence of marine macroalgae at the low-salinity end of the gradient.

Although seaweeds are well able to tolerate short-term fluctuations in salinity [13], their growth may be inhibited when they are exposed to low salinity for extended periods [14–17] or when they are faced with additional stressors close to their lower salinity limit [18]. One of the main effects of salinity stress on seaweed physiology is the formation of reactive oxygen species (ROS), which are synthesised in response to different stressors and may lead to cellular damage by causing oxidative stress [19]. The production of antioxidant compounds is one of the most important and common components of the stress response to ROS [20, 21]. Hyposalinity stress in macroalgae may also lead to the inhibition of photosynthesis [22], with potential negative repercussions for the balance between photosynthetic activity and respiration, which plays a central role in algal physiology. These salinity responses, however, are inconsistent among seaweeds, with some algae showing short-term patterns of increased respiration and either inhibition or enhancement of photosynthetic activity (reviewed in Karsten et al. [6]). Other responses to changes in salinity include the activation of mechanisms to maintain constant cell turgor through managing the concentration of osmolytes such as inorganic ions and organic compounds in the cytoplasm and the vacuoles [22]. In seaweed cells, ion concentration is controlled by actively importing ions from a hyposaline environment or excreting them in a hypersaline environment via ion channels and ion transporters [23]. As the flux of inorganic ions may result in metabolic oxidative damage to intracellular components, this osmotic strategy is combined with the accumulation of organic osmolytes [24], which, in seaweeds, are often carbohydrate by-products of photosynthetic activity [22].

In the brackish water of the Baltic Sea, *Fucus vesiculosus* is the dominant brown seaweed and the major foundation species in rocky littoral shores [25]. The species has a broad tolerance range to salinity, with populations stretching from the highly saline waters of the Baltic Sea entrance (24 PSU) to the relatively hyposaline waters (2 PSU) of the northern and eastern margins of the Baltic Sea and the White Sea. Low salinity limits the species’ distribution most probably due to the low tolerance threshold of the gametes [26]. However, Baltic populations of *F. vesiculosus* have evolved a broader tolerance to low salinity than their Atlantic counterparts [27]. Furthermore, both Atlantic and Baltic Sea populations are locally adapted to salinity; a reciprocal transplant experiment in a common garden found that populations grew better in their local salinity compared to the foreign salinity (24 and 4 PSU, respectively [28];). Despite such adaptation the Baltic Sea salinity gradient remains as a major factor affecting size, morphology and chemical contents of *F. vesiculosus* [29]*.*

The physiological mechanisms underpinning the osmoregulatory abilities of *F. vesiculosus* are still unknown. Without this information, it is difficult to predict how this species may react to the further decrease in salinity that has been projected for the Baltic Sea as a result of climate change. Here, we studied the process of hyposalinity acclimation by quantifying differences in gene expression between algae in current ambient salinity and those in future hyposaline conditions, which were predicted by a recent climate model [12]. In order to determine the functions of differentially expressed genes, given that no reference transcriptome was previously available for the species, we assembled de novo and annotated a full transcriptome library for *F. vesiculosus*. Because the species shows both strong genetic structuring [30] and geographic variation in salinity tolerance [10], we also assessed possible among-population variation in gene expression by including algal specimens from two geographic localities.

## Results

### Transcriptome assembly and annotation

After the quality filtering, we retained 88.7% of read pairs (22.6 million), or 52.7 to 95.7% per sample (mean ± SE; Current condition: Rauma = 17.8 ± 1.5; Parainen = 15 ± 3.2, Future condition: Rauma = 15.3 ± 1.8, Parainen = 17.5 ± 0.7, Table 1). De novo assembly with the default Trinity parameters produced 295,013 genes and 382,992 transcripts (Table 2). Filtering with TransRate and TransDecoder reduced the size of the assembly to 33,487 genes and 58,943 transcripts (Table 2). According to the BUSCO assessment, our assembly is 89.4% complete, i.e. it contains 271 complete single-copy and 163 complete duplicated reference eukaryotic genes, while 19 genes are fragmented and 13 are missing. Of 24,486 *E. siliculosus* reference proteins, 43% had a Conditional Reciprocal Best Blast in our *F. vesiculosus* assembly.

**Table 1:** The number of reads (total million read pairs), the quality score (Q30), and the % of read pairs successfully mapped to the final transcriptome assembly in the gene expression analysis for each of the samples sequenced

Sample	Population	Climate condition	Reads (M) row data	Reads (M) filtered	> = Q30	% Reads mapped
R1	Rauma	Future	15.68	14.3	89.9	34.2%
R1	Rauma	Current	23.43	21.05	91.9	46.5%
R3	Rauma	Future	24.02	20.82	89.8	48.5%
R3	Rauma	Current	15.53	14.14	89.1	41.03%
R7	Rauma	Future	13.71	12.46	90.1	38.7%
R7	Rauma	Current	17.68	16.92	91.0	42.5%
R9	Rauma	Future	14.87	13.63	89.7	41.1%
R9	Rauma	Current	20.7	18.99	91.5	44.8%
S1	Parainen	Future	17.62	16.38	89.1	37.9%
S1	Parainen	Current	11.98	11.08	89.7	39%
S8	Parainen	Future	20.21	18.88	89.5	37.8%
S8	Parainen	Current	24.16	21.34	91.7	44.6%
S9	Parainen	Future	18.58	17.34	89.9	46.5%
S9	Parainen	Current	23.30	12.28	90.5	46%

**Table 2:** Summary statistics for the transcriptome assembly. The evaluation of the assembly is shown both for the original assembly and after filtering with TransRate and TransDecoder

Transcriptome assembly	Original assembly	Filtered assembly
Total # genes	295,013	33,487
Total # of transcripts	382,992	58,943
N50 transcript size, bp	854	1206
Average transcript length, bp	581.5	912
Total assembled bases	222,713,028	53,753,658

**Fig. 1:**
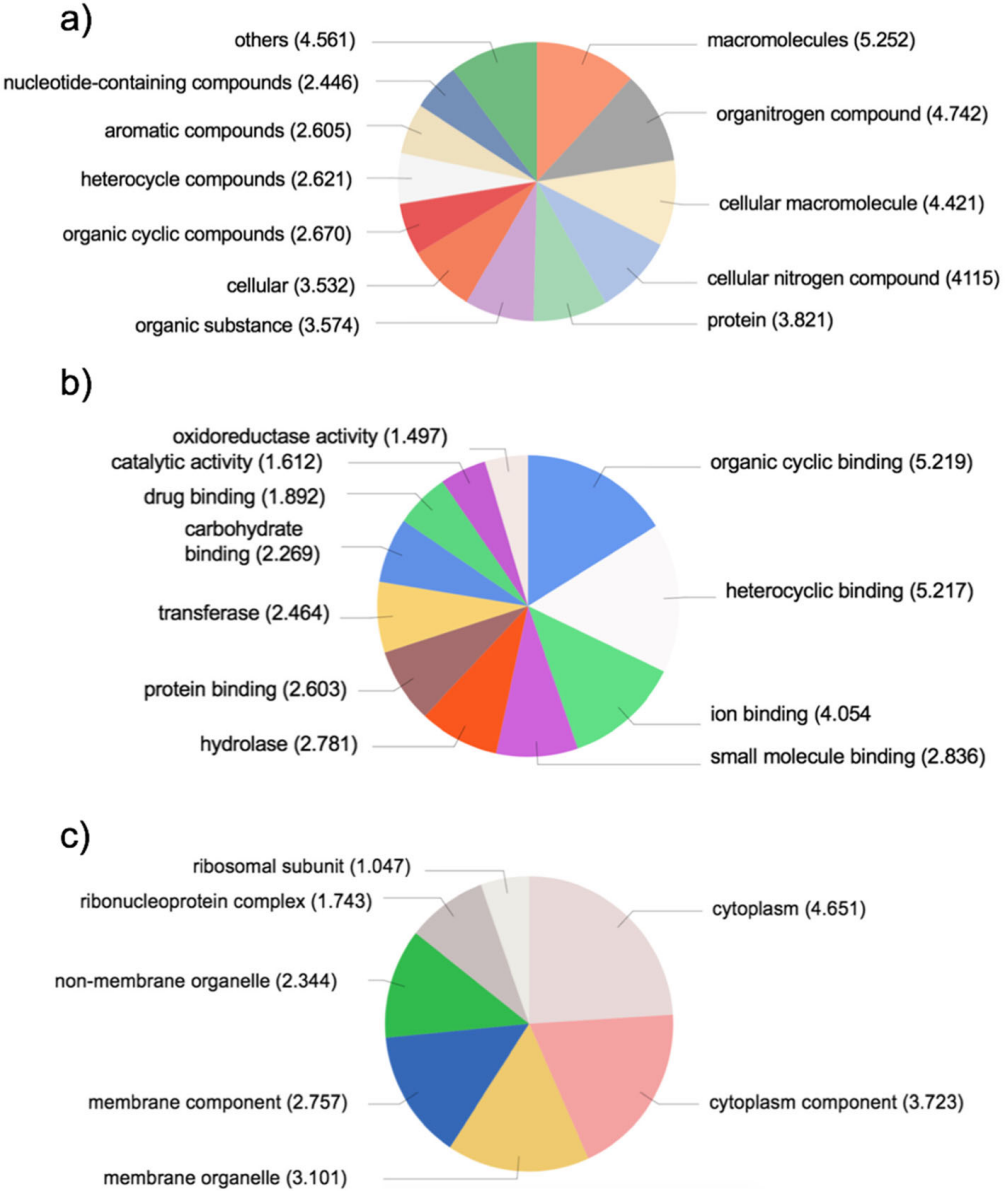
Annotation summary of de novo transcriptome assembly of *F. vesiculosus*: GO categories for biological processes (**a**), molecular functions (**b**), and cellular components (**c**). Numbers in brackets indicate the number of genes belonging to each group

​ In the assembly, 16,195 genes (48%) could be assigned one or more GO mapping terms. In total 57,220 annotations were assigned with mean GO level = 6.69. In the GO classification by Biological Process, the largest group was related to translation, similar to what was found in *F. vesiculosus* transcriptome analysis by Martins et al. [31]. Otherwise, the *Fucus* transcriptome represents a wide range of biological processes, none of them being particularly dominating (Fig. 1a). In the GO classification by Molecular Function, the largest groups were related to the binding of organic cyclic compounds and heterocyclic compounds, followed by ion binding (Fig. 1b). Finally, in the GO classification by Cellular Component, the transcripts representing all main cell components, the largest groups related to cytoplasm (Fig. 1c).

KEGG metabolic pathways provide another way to summarize functional content of the expressed genes. In our assembly, the ten most highly represented molecular functions (as defined by the number of sequences mapped to pathway) were purine and thiamine metabolism, biosynthesis of antibiotics, aminobenzoate degradation, pyrimidine metabolism, glycolysis and glucogenesis, carbon fixation in photosynthetic organisms, phenylpropanoid biosynthesis, drug metabolism and amino sugar, nucleotide sugar metabolism (see Additional file 1 for a list of total 133 pathways found in the assembly).

### Variation in gene expression between populations in response to hyposalinity

We were able to map 34–46% of reads per sample to the final transcriptome assembly, for a total of 33,487 genes and 58,943 isoforms (Tables 1, 2). A paired t-test showed that there was no significant difference in mapping success between treatments (t = 1.21, *p* = 0.273). Of all the genes, 32,345 were expressed at the level of at least one transcript per million in at least one sample in the expression matrix.

**Fig. 2:**
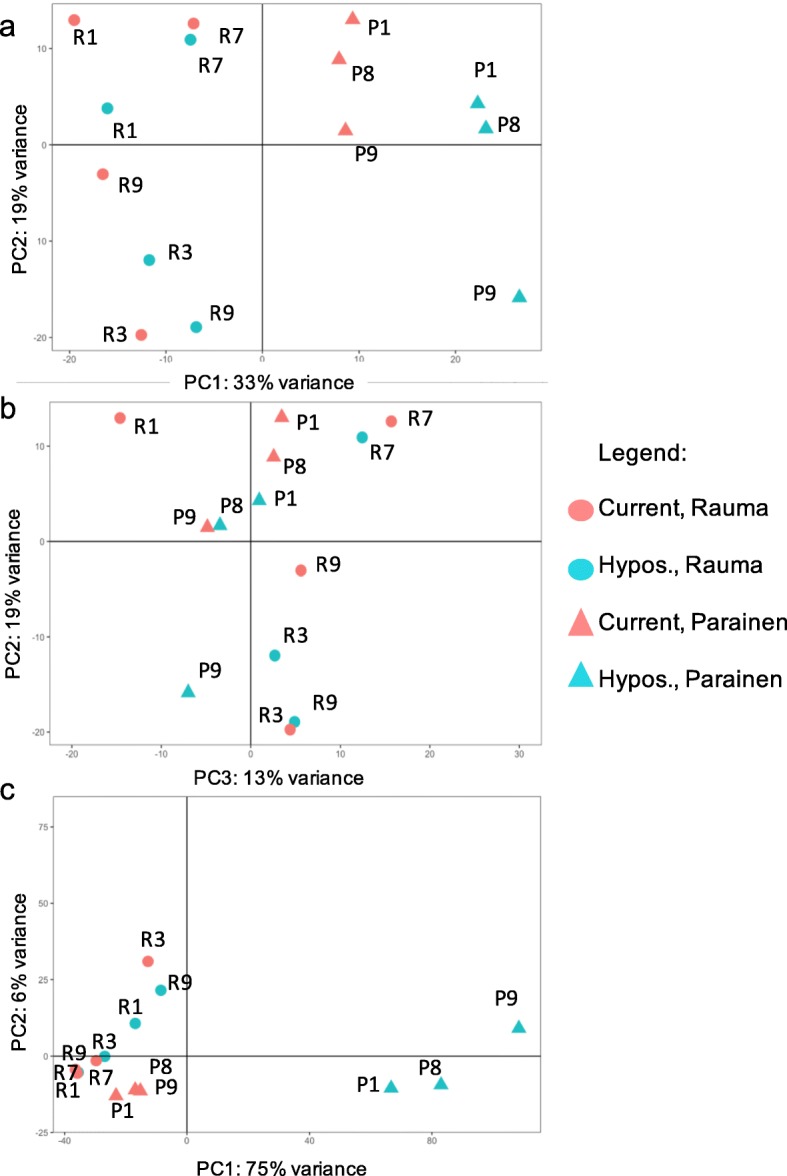
Principal component plot of *F. vesiculosus* samples based on their gene expression profiles. The identity of each sample is indicated by the code next to the dot/triangle representing it. Genes were grouped using PCA (Principal Component Analysis) based on the pairwise distances between the populations and treatments that were in turn based on **a**) and **b**) the normalised read count from all genes as a proxy for the biological coefficient of variation, and **c**) the normalised read count from the differentially regulated genes (*P* < 0.05, absolute value of the fold change > 2)

Principal components suggested that the algal populations differed in their response to hyposalinity (Fig. 2a, b and c). The analysis of similarity using the most variable genes, which detected differences in gene expression between populations (R = 0.783, *P* = 0.001) and between salinity treatments (R = 0.51, *P* < 0.01), confirmed the pattern: we observed a similar pattern of gene expression in both populations in present conditions and in the Rauma population in future conditions, while the Parainen population had a unique gene expression pattern when exposed to hyposalinity (Fig. 2a, b and c). Because of the differences between populations, we ran the downstream analysis separately for each population.

**Fig. 3:**
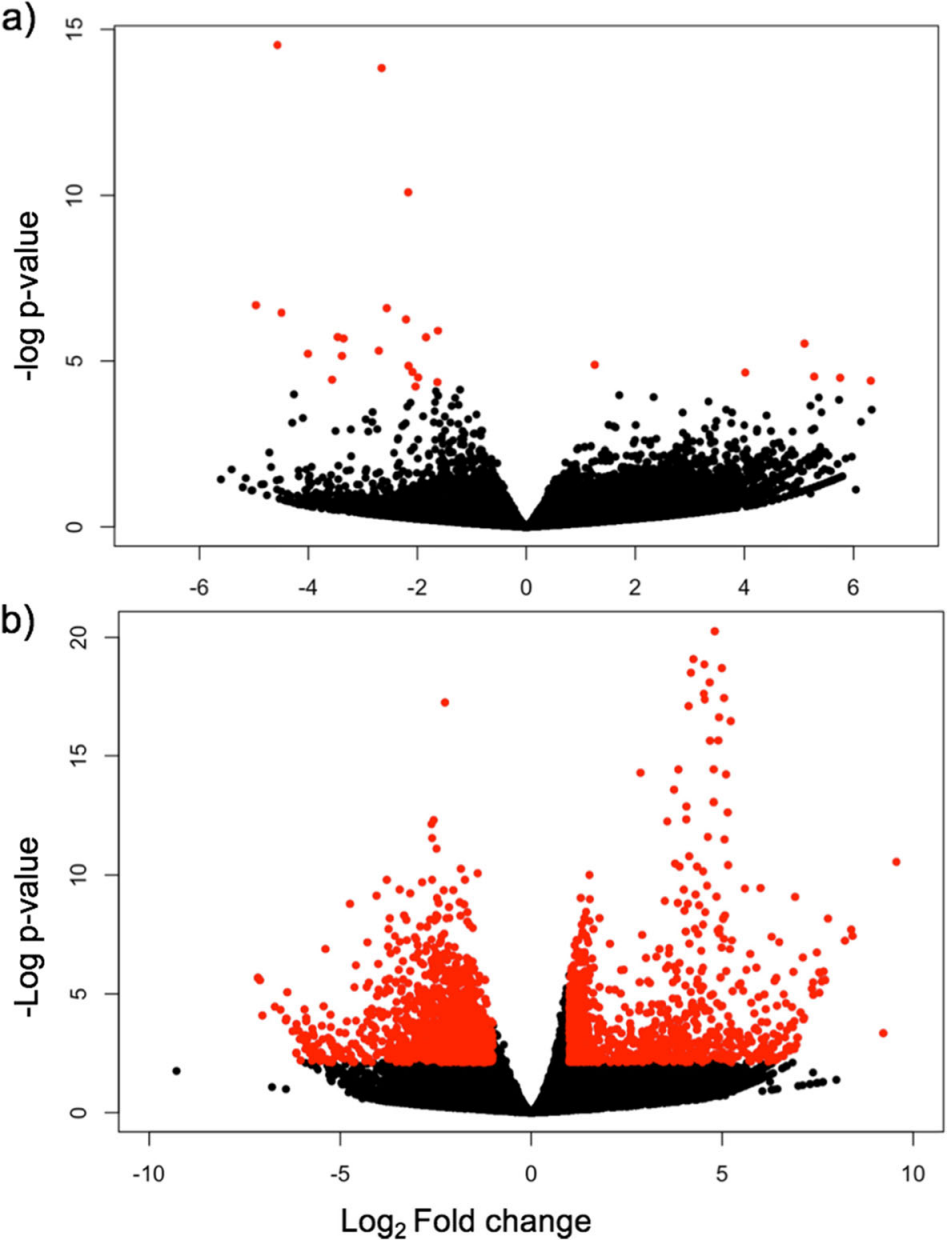
Volcano plots showing genes differentially expressed between salinity treatments and the magnitude of the expression change for Rauma (**a**) and Parainen (**b**) populations. Each point represents one of 33.487 genes. The x-axis shows the log_2_ fold change and the y-axis shows log_2_ *p*-value, adjusted for multiple comparisons. Differentially expressed genes at adjusted p-value < 0.05 and absolute log_2_ fold-change > 1 are indicated in red

We found a very weak response to hyposalinity in algae from the Rauma population: when we applied the criteria FDR < 0.05 and |log_2_FC| > 1, only six genes were upregulated and 20 were downregulated (Fig. 3a). All of these genes were also differentially expressed in the Parainen population in response to hyposalinity, and two genes in particular had very similar responses in both populations: a glucose/sorbonose dehydrogenase and a thiosulfate sulfurtransferase (Additional file 1, and Additional file 2). In contrast with the weak response in the Rauma population, the expression response to hyposalinity was very pronounced in the Parainen population: a total of 3072 genes were differentially expressed between the salinity conditions. Among these, 1633 genes were upregulated and 1439 were downregulated in hyposaline conditions (FDR < 0.05, |log_2_FC| > 1, Fig. 3b).

### Annotation of the highly responsive genes

To identify the mechanisms behind the stress response, we annotated the genes in the Parainen population that had the largest expression changes in response to hyposalinity (i.e. |log_2_FC| > 2). This yielded 399 and 396 up- and down-regulated genes, respectively. We grouped the most-relevant genes according to their putative functions: the response to oxidative stress (Table 3), membrane/cytoskeleton composition and transport (Table 4), and energy production and conversion (Table 5).

**Table 3:** Genes involved in the oxidative stress response that were differentially expressed in future vs. present salinity conditions in *F. vesiculosus*

Regulatory response to future salinity	Gene annotation	# of genes	GO term Biological process
up	glutathione reductase	1	GO:0045454 cell redox homeostasis
	disulfide isomerase	4	GO:0004362
	nucleoredoxin-like	2	GO:0045454
	ribulose-1,5-bisphosphate carboxylase/oxygenase	1	GO:0055114
	superoxide dismutase	2	GO:0019430
	vanadium-dependent bromoperoxidase 2	2	GO:0055114
	14–3-3 protein	7	GO:0055114
	hydroxyphenylpyruvate dioxygenase	4	GO:0055114
	copper oxydase	1	GO:0016491
	glyceraldehyde-3-phosphate dehydrogenase	3	GO:0055114
	methylmalonate-semialdehyde dehydrogenase	1	GO:0055114
	pyruvate dehydrogenase	1	GO:0055114
	heat shock proteins	3	GO:0006950
down	phenylacetate-CoA oxygenase	1	GO:0016491
	stearoyl-CoA desaturase	1	GO:0016491
	xanthine dehydrogenase	1	GO:0016614
	NAD(P)/FAD-dependent oxidoreductase	1	GO:0055114

**Table 4:** Genes involved in membrane/cytoskeleton composition and transport that were differentially expressed in future vs. present salinity conditions in *F. vesiculosus*

Regulatory response to future salinity	Category	Gene annotation	# of genes	GO term Biological process
up	membrane and cytoskeleton composition	mannuroan C-5-epimerase	2	GO:0016021
		acetyl-COA carboxylase	1	GO:0003989
		actin	2	GO:0006972
up	transmembrane transport	ATPase	1	GO:0042626
		ADP/ATP translocase	1	GO:0046902
		ankyrin	9	GO:0006357
down	membrane and cytoskeleton composition	mannuronan C-5-epimerase	9	GO:0016021
		tubulin proteins (α, β)	17	GO:0005200 GO:0007010
down	transmembrane transport	cation diffusion Facilitator	1	GO:0008324
		voltage-gated Ion Channel	2	GO:0006813
		ABC transporter	2	GO:0055085

**Table 5:** Genes involved in energy production and conversion that were differentially expressed in future vs. present salinity conditions in *F. vesiculosus*

Regulatory response to future salinity	Category	Gene annotation	# of genes	GO term Biological process
up	photosynthesis regulation	cell division protein FtsH	1	GO:0010205
		fucoxanthin proteins	23	GO:0031409
		light harvesting	5	GO:0009768
		photosystem II reaction center protein	2	GO:0016682
		photosystem I P700 apoprotein A2	1	GO:0022900
		oxygen-evolving enhancer protein	2	GO:0042549
up	ATP synthesis	ATP synthase	6	GO:0045261
up	respiration	cytochrome b-c1 complex	2	GO:0005750
down	metabolism of carbohydrate	beta-glucanase, GH16 family	1	GO:0005975
		glucose dehydrogenase	2	GO:0047936
		UTP-glucose-1-phosphate uridylyltransferase	1	GO:0006011
		glutamine-fructose-6-phosphate transaminase	2	GO:0006002
		CAMK protein kinase	5	GO:0035556
down	Metabolism of amino acids	glutamate cysteine ligase	1	GO:0006750
		glutamate receptor ionotropic, kainate 2-like	1	GO:0035235
		glutaredoxin	1	GO:0045454

**Fig. 4:**
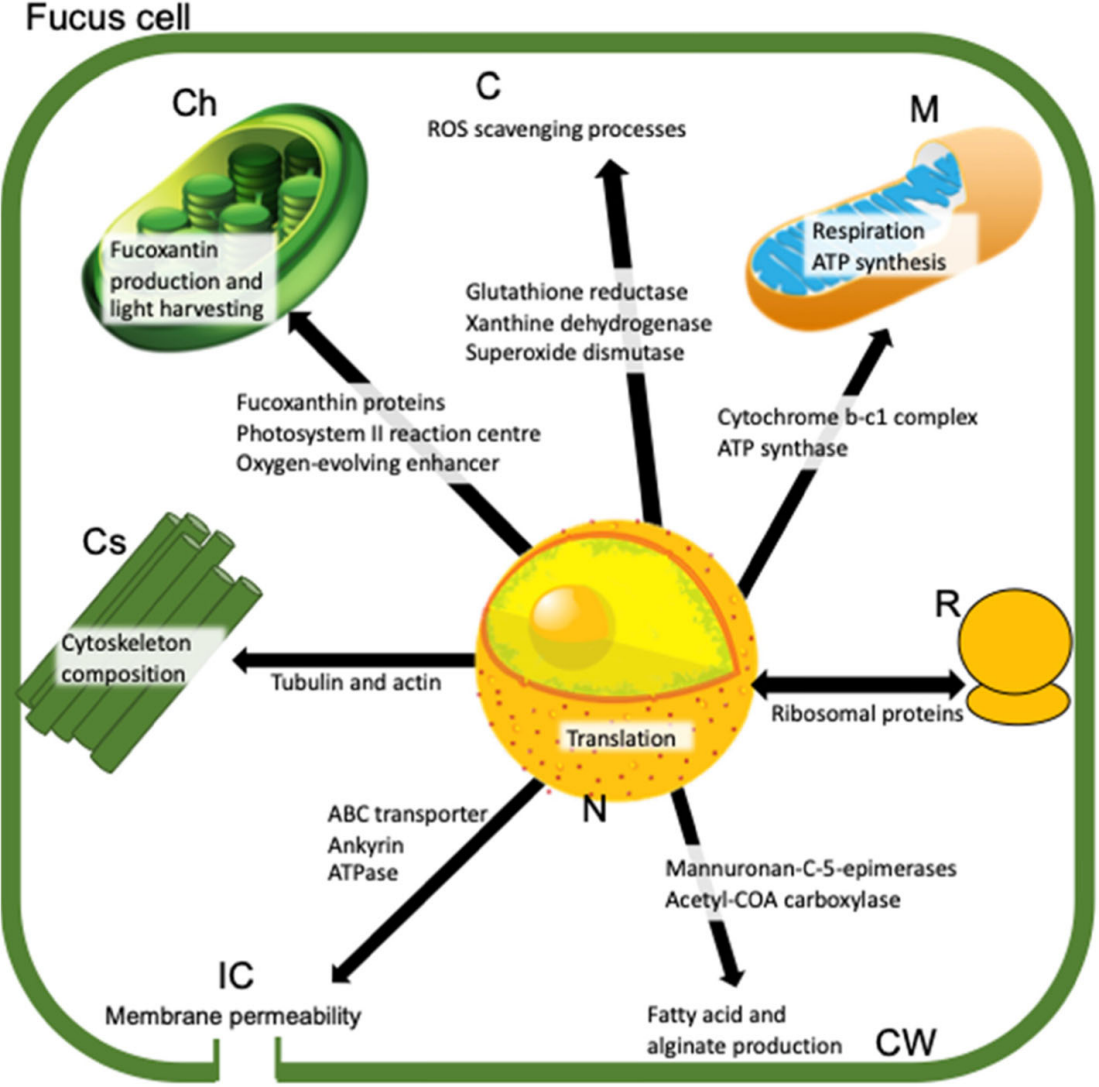
Schematic depiction of the main proteins involved into the differentially expressed genes as response to hyposalinity and the main processes involved inside a *Fucus* cell. Each cell component is represented only once due to space availability and its name coded as follows: (C = Cytosol, Ch = Chloroplast, Cs = Cytoskeleton, CW = Cell Wall, IC = Ion Channel, M = Mitochondrion, R = Ribosome, N = Nucleus)

Hyposalinity induced the upregulation of at least 32 genes directly involved in defensive responses to oxidative stress (Table 3, Fig. 4). Among these, we found genes coding for enzymes that are used as defence against cell damage from free radicals, such as glutathione reductase, superoxide dismutase, disulfide isomerase, nucleoredoxin-like protein, and vanadium-dependent bromoperoxidase 2. Seven of these genes have an important role in maintaining the osmotic balance of the cell. Finally, three of the upregulated genes encoded heat-shock proteins. Instead, only four genes related to oxidative stress were downregulated. Among these was xanthine dehydrogenase, whose function includes the replacement of monounsaturated fatty acids that are turned into polyunsaturated fatty acids by oxygen radicals.

Hyposalinity triggered the differential gene expression of at least 32 genes that control the composition of the membrane and cytoskeleton. Among these, mannuronan C-5-epimerases were both down- and up-regulated. The upregulated acetyl-COA carboxylase and actin protein directly affect cytoskeletoncomposition as they, together with ATP binding transporters such as ATPase and ATP translocase, are responsible for the stability of the membrane. Nine genes containing an ankyrin domain were also upregulated; these coded for proteins with functions ranging from ion transport to the transmembrane transit of ions and molecules. Instead, other cytoskeleton components, such as tubulin proteins, were downregulated, as were genes that control ion channels, such as voltage-gated ion channels and the ABC transporter.

Hyposalinity triggered the upregulation of several genes involved in energy production and conversion, whose functions were implicated in the regulation of photosynthesis, ATP synthesis, and respiration (Table 5, Fig. 4). Among the 34 genes that regulate photosynthesis, 23 coded for fucoxanthin, a xanthophyll pigment that shades the photosystems from high irradiance. Five more genes coded for proteins that protect and repair photosystems I and II from oxidative stress, and five others for proteins that regulate the transfer of energy in the reaction centre of the photosystems. The regulation of the ATP cycle was represented by six genes implicated in ATP synthesis, and two genes involved in the respiratory chain in mitochondria, both coding for cytochrome b-c1. A total of 11 genes that participate in the metabolism of carbohydrates and proteins, including the decomposition of glucan and glucose, were downregulated in response to hyposalinity. The activation of enzymes that regulate glycogen metabolism was inhibited through the downregulation of at least five protein kinases (CAMK). Furthermore, the synthesis of glutamate, an amino-acid precursor of glutathione, was reduced through the downregulation of glutamate cysteine ligase, ionotropic glutamate receptor, and glutaredoxin.

In addition to these functional groups, hyposalinity triggered expression changes in several protein families with very broad functions, such as ribosomal proteins. For example, 55 upregulated genes code for ribosomal subunits 40S and 60S, which act in DNA repair and protein translation. For other genes the exact function in brown algae is unknown. The complete list of genes whose expression was affected by hyposalinity in the Parainen population can be found in Additional file 1.

### Comparison of hyposalinity response in Fucus to other brown algae

Dittami et al. [32] reported 161 annotated genes responding to hyposalinity stress in *Ectocarpus*. The majority of these DE genes (134 out of 161) were found among DE genes in *Fucus,* resulting in 78 unique hits (Additional file 3). These hits include genes belonging to the biological processes influenced under hyposalinity conditions in *Ectocarpus*, such as amino acid metabolism, photosynthesis, transport, carbohydrate metabolism, protein turnover, general stress response, and regulation of transcription and translation. Similar search was performed for 230 unknown *Ectocarpus* genes responding to hyposalinity stress. Of them, 48 genes showed sequence similarity to differentially regulated genes in *Fucus.* Among those, 30 genes have some functional information in *Fucus* (Additional file 3), while 18 genes remain unknown. In *Sargassum*, Qian et al. [33] reported 34 proteins involved in hyposalinity response, of them, 25 showed similarity to *Fucus* DE genes, resulting in 17 unique hits. These hits included genes involved in photosynthesis, carbohydrate metabolism, energy metabolism, cytoskeleton and protein folding (Additional file 3). In summary, we found considerable consistency among the brown algal species in their gene responses to hyposalinity, but also some taxon specificity.

### Genotypes of the individuals in the experiment and fixed differences between the populations

After filtering, we retained 260,571 bi-allelic SNPs. Both in the PCA and the clustering analyses 6 of 7 individuals were well separated along while two individuals from Rauma remained close, suggesting that they may be clones of the same genotype (Additional file 4). We also observed the separation by population along the first PCA axis and the clustering by population in the NJ-tree (Additional file 5). We found a total of 10,241 sites fixed for different alleles in the two populations. In both populations, the numbers of differentially expressed genes and the genes with fixed differences were highly dependent (contingency table tests; Parainen: G^2^ = 935, DF = 1, *p* < 0.001; Rauma: G^2^ = 6.1, DF =, *p* < 0.05). We found that in the Parainen population28.0% of non-DE genes contained fixed sites, with on average 2.16 ± 0.05 fixed SNPs (mean ± C. I.) per gene. In Rauma, 30.6% of non-DE genes had such sites, with on average 2.15 ± 0.04 (mean ± C. I.) fixed SNPs per gene. Among DE genes from Parainen sample 1715 (55.9%) genes contained fixed sites a, with an average 2.2 ± 0.11(mean ± C. I.) SNPs per gene. For Rauma, 14 (53.8%) DE genes contained the fixed sites with 2.4 ± 1.5 fixed SNPs (mean ± C. I.). Thus, the proportion of genes with the fixed differences in the two populations were higher in DE genes than in non-DE genes, while the average number of fixed sites per gene seems to be similar in DE and non-DE genes.

## Discussion

### De novo assembly of *Fucus vesiculosus* transcriptome

In this study we produced a de novo transcriptome, containing 33,487 assembled “genes”. It is reasonably complete, i.e. contains 89% of the conserved eukaryotic genes. Despite the filtering, this assembly is still somewhat redundant, as suggested by found duplicated BUSCO-orthologs, and likely overestimates the number of genes. Of the *Ectocarpus* reference genes, 43% appeared to have one-to-one orthologs in our *F. vesiculosus* assembly, which may be expected given a large evolutionary distance between Fucales and Ectocarpales [34].

The most common gene functions in the characterized transcriptome were either core eukaryotic metabolic pathways (e.g. purine, thiamine and pyrimidine metabolism and glycolysis), or pathways characteristic for plants and algae, such as carbon fixation or metabolism of amino sugars and nucleotide sugars (sugar donor for various glycans, and also connected to fructose and mannose metabolism). Another highly represented global pathway was biosynthesis of antibiotics, which includes biosynthesis of carbohydrates, aromatic amino acids (shikimate pathway) and secondary metabolites and is represented by many genes in e.g. green algae. Finding the “Drug metabolism – other enzymes” pathway among most highly represented may appear somewhat unexpected. *Fucus* genes belong to the part of the map referring to degradation of anti-cancer and immunosuppressive agents: irinotecan, fluorouracil, isoniazid, azathioprine and 6-mercaptipurine. Interestingly, sulfated polysaccharides from *F. vesiculosus* (fucoidan) and other algae are increasingly used in cancer therapy together with these drugs to mitigate their toxic side effects, and even themselves are suggested to have antitumor effects. Finally, another unexpected finding was several genes from phenylpropanoid biosynthesis pathway, leading to lignin biosynthesis in higher plants (Embryophyta), i.e. cellobiase, cinnamyl alcohol dehydrogenase, caffeic acid O-methyltransferase and lactoperoxidase. For long time lignin was thought to be a key innovation in higher plants, but recently was also found in the closest relative of higher plants, streptophyte algae [35], and also in red algae [36]. Genes from the lignin biosynthesis pathway were also found in genomes or transcriptomes of diatoms and other member of Stramenopiles [37], but it is unclear whether this group can perform the final step of lignin polymer biosynthesis. In light of this, it is interesting that in *Fucus* transcriptome we found lactoperoxidase, responsible for the last steps of lignin synthesis from coniferyl alcohol or p-coumaryl alcohol.

### Among-population variation in hyposalinity tolerance

Here, we show that the physiological acclimation to hyposalinity differed between the two populations of *F. vesiculosus*. The Rauma population showed hardly any expression response, while in the Parainen population more than 3000 genes responded to the change in salinity. We emphasise that our hyposalinity manipulation took place gradually and the algae had several days to acclimate to the slow decrease in salinity. In nature, salinity fluctuates during rainy periods, when river runoff is directed at rockweed stands, or when freshwater runoff spreads under ice. Because the salinity change in this experiment was so gradual, we do not believe that the expression response in our experiment represents acute hyposalinity shock, i.e. a direct stress response that would be expected if an alga is suddenly exposed to hyposaline conditions. Instead, we interpret the expression changes as representing the acclimation process, i.e. the adaptive plasticity that allows algae to sustain their functions across variable conditions, with the among-population differences that we observed indicating the presence of geographic differentiation among populations in their acclimation ability. Differentiation in tolerance to abiotic stressors has been documented previously for Baltic *F. vesiculosus*: a study of algal performance in expected future conditions of hyposalinity and warming found different responses in populations from the Bothnian Sea with respect to those from the Archipelago Sea [10]. The among-population variation found here may arise as a consequence of population-level adaptation to local salinity. Indeed, long term yearly measurements (from 1900 to 2005) indicate that Rauma and Parainen differ for the mean salinity but most importantly for its variation [Rauma: mean = 5.25, max = 6.2, min. = 1; Parainen: mean = 6.06, max = 6.4, min. = 5.3, 59]. This difference in both the mean and variation of salinity could be biologically highly relevant because of its implications for the populations’ salinity tolerance limits. Thus, the Rauma population may be better adapted to low salinity as it experiences higher variation throughout the year than the Parainen population, and it is perhaps better adapted to more extreme fluctuations in salinity.

However, differences between the Rauma and Parainen populations were not created by the constitutive upregulation of genes involved in the acclimation process, as has been suggested for some resilient coral species (so-called “constitutive frontloading gene expression” [38];), because the populations did not differ in their gene expression patterns under ambient conditions. Instead, the possible mechanism could be a local adaptation, i.e. genetic changes of some other traits that no longer respond plastically to hyposalinity in Rauma. Local adaptation to salinity conditions may be particularly likely in the Baltic Sea because of the presence of a strong salinity gradient. There is already evidence for regional adaptation in Baltic Sea populations with respect to those in the North Atlantic [28], but data at a smaller geographic scale within the Baltic Sea are lacking. However, several characteristics of *F. vesiculosus* – the strong genetic structuring of populations [30], very limited gamete dispersal ability [26], and the potential for gene flow through floating dispersal [39] – generate promising conditions for the evolution and maintenance of local adaptations. In the present study we found that the proportion of genes fixed for different alleles in the two populations was higher in DE genes compared to non-DE genes, which suggests local adaptation. Conducting a genomic scan of these and other local populations of *Fucus* in the Baltic will be a focus of future research.

### Mechanisms of hyposalinity acclimation

Our study describes for the first time differential expression of genes in response to hyposalinity in the brown seaweed *F. vesiculosus*, based on the performance of the Parainen population. In general, we found large overlap between genes differentially regulated in hyposalinity in *Fucus* and in the two other studied brown alga: *Ectocarpus* and *Sargassum* [40, 41] They are involved in such biological processes as photosynthesis and energy production; cytoskeleton and membrane transport; and stress response. These are discussed in details below.

Of particular interest are the genes related to the oxidative stress response, which defends cells from oxidative bursts that occur due to an excess of ROS. Among the upregulated genes found here, we observed antioxidant enzymes such as superoxide dismutase and glutathione reductase, which are a commonly found components of the stress response [21]. In addition, we found genes coding for disulfide isomerase and nucleoredoxin-like proteins, which are involved in the production of thioredoxins, low-molecular weight proteins involved in the regulation of enzymatic redox reactions in the chloroplast [32, 42]. Another antioxidant, vanadium-dependent bromoperoxidase 2, plays an important role in the salinity tolerance of brown algae by contributing to cell strengthening [43]. This stress gene has been previously identified in protoplasts of *L. digitata,* and it is involved into the synthesis of halo-ganic compounds possibly linked to defences against pathogens and scavenging H_2_O_2_ [44]. This compound also contributes to the production of secondary compounds such as phlorotannins [40], which are known to increase as a response to biotic and abiotic stress [45, 46]. Certain heat shock proteins (HSPs) were also upregulated. In many organisms as well as in brown and red algae [40, 47], the upregulation of HSPs is linked to abiotic shifts because these proteins stabilise other proteins and cell membrane structure, thereby improving cellular homeostasis [48]. Interestingly, we also detected the downregulation of genes related to the ROS scavenging mechanism. Among these was a gene coding for xanthine dehydrogenase, an enzyme that controls the metabolism of purines and pyrimidines [33] and which is upregulated as a response to desiccation in *Arabidopsis spp*. [49]. Since both desiccation and salinity affect intracellular osmotic potential and turgor, the downregulation of these genes here may indicate an attempt to remove excess intracellular water due to hyposalinity.

### Changes in membrane and cytoskeleton structure

Gene expression changes indicated the importance of membrane and cytoskeleton modifications as a response to hyposalinity. Specifically, the change in regulation of genes encoding mannuronan-C-5-epimerases indicates that alginates have a key role in hyposalinity acclimation*.* Alginates are the main polysaccharide components of the cell walls of brown algae, and they can form almost half of algal dry weight [50]. These polysaccharides are important in acclimation because they separate the cell from the surrounding environment, which is likely to be especially important when the osmotic environment becomes challenging (reviewed in [43]). The upregulation of alginate-related genes as a response to hyposalinity has also been reported in other brown algae [41].

The upregulation of acetyl-COA carboxylase has implications for the biosynthesis of fatty acids [51], which are important membrane components. Fatty acids can become the targets of ROS, which exchange a hydrogen with them and thus compromise their stability [45]. Since hyposalinity generates oxidative stress, increased production of fatty acids may be needed to maintain the membrane stability. In addition, most of the genes in this functional group were linked with actin and tubulin production, and their expression was inversely affected, with the former being upregulated and the latter downregulated. A similar pattern was described in the brown alga *S. fusiforme* [41]. Both actin and tubulin play important roles in determining the strength of the cytoskeleton and cell volume [52]. The production of ROS is known to induce reactions such as oxidative modification, acetylation, and phosphorylation in tubulin polymers in the cytoskeleton in response to stress such as a change in water salinity [53]. Our result likewise indicates that changes in actin and tubulin production are involved in hyposalinity acclimation, possibly because of the oxidative stress involved.

The regulation of membrane-bound ion channels balances the amount of ions transported in and out of cells [54]. Here, certain transportation-related genes were upregulated, such as ATPase which is known to respond to hyposalinity (reviewed by [6, 47]). Several genes coding for ankyrin proteins were also upregulated. Proteins with ankyrin motifs serve many different biological functions, including controlling vesicular trafficking [55], which is involved in the hyposalinity response [40]. In seaweeds, the concentration of intracellular ions is also controlled by ion-selective carriers that are activated by the membrane potential [23]. We found that genes coding for voltage-gated ion channels, which regulate the membrane permeability for sodium and potassium [56], were downregulated in hyposaline conditions. Downregulation of these genes may help to balance intracellular ion composition under conditions of osmotic imbalance. Furthermore, we also found downregulation in two ABC transporter-related genes. ABC transporters detoxify the cell through the excretion of toxic compounds and regulation of intracellular ion concentration [57]. Our results suggest that this transporter may have a function in hyposalinity acclimation in *F. vesiculosus,* as has been suggested to be the case in some seaweeds [58]*.*

### Energy production and conversion

Photosynthetic activity is fundamentally important for seaweeds, but can become inhibited under stressful conditions such as hypo/hypersalinity, high irradiance, and herbivory [40, 59–61]. Our study showed that hyposaline conditions caused the upregulation of genes that control the production of fucoxanthin and other light-harvesting proteins that bind to photosynthetic pigments. Fucoxanthin is among the most abundant carotenoids in seaweeds and is responsible for their brownish colouration and the protection of the photosystemic apparatus from excess of light [62]. Inhibition of the photosystem in stressful conditions may provide several benefits. First, since the production of photosynthetic proteins requires energy, photoinhibition may make available additional resources for osmotic adjustments*,* thus reducing the overall cost of acclimation. This strategy was proposed to explain the downregulation of chloropyll a/c binding proteins of *E. siliculosus* acclimating to hypo- and hyper-salinity as well as *Chaetoceros neogracile* acclimating to thermal stress [63]. A reduction in primary metabolism, such as photosynthetic activity, may be a way to save energy [40]. In addition, the photosynthetic machinery usually absorbs more photons than necessary, and then dissipates the excess by rearranging photosynthetic pigments (e.g., from a single chlorophyll to a triple chlorophyll). This chlorophyll reacts with oxygen, generating ROS, and thus, oxidative stress. Such reduction in the electrons reaching the reaction centres would translate into a lower need for the synthesis of new ROS [64].

When *F. vesiculosus* was exposed to hyposalinity, energy production was enhanced via the upregulation of genes involved in respiration and ATP synthesis. The balance between respiration and photosynthesis plays a crucial role in algal physiology, as it determines growth and shapes competition for irradiance and resources [58]. An imbalance between these two processes, caused by abiotic or biotic stressors, may lead to a decrease or cessation in growth or, in extreme cases, death [65]. Respiration enables the storage of biochemical energy as ATP, so respiration and ATP metabolism are strongly correlated. The upregulation of respiration that we observed as a response to hyposalinity likely serves to produce the energy required for osmotic adjustments.

Compared to respiration and ATP synthesis, we detected the opposite pattern for carbohydrate metabolism, with six genes downregulated. A similar response was also reported in *E. siliculosus* as a response to hyposalinity [40]. Carbohydrate synthesis in autotrophs is highly dependent on photosynthetic activity, so it is probable that this decrease in carbohydrate metabolism is a consequence of the photoinhibition caused by hyposalinity stress. It is also worth noting that carbohydrates can be used for osmotic adjustments by means of their accumulation in vacuoles or excretion through the cell membrane [22]. In brown algae, a previous study indicated that the concentration of mannitol, the main carbon storage compounds in brown algae, varies according to the sea water salinity, and it is recognised as part of the osmotic adjustments in the algal cell [66, 67]. Previous studies have shown that Baltic *F. vesiculosus* differs from its Atlantic counterpart by having lower photosynthetic activity, higher respiration rate [68], and lower concentrations of mannitol, the main carbohydrate used for energy storage [53, 55, 56]. Our results may indicate that the differential expression of genes involved in the above processes may be attributed to its adaptation to the brackish water of the Baltic Sea, and, furthermore, that these modifications may be among the first and perhaps most important adjustments behind hyposalinity acclimation in this species.

Hyposalinity hindered glutathione synthesis by suppressing the expression of early steps of the glutamate pathway. In seaweeds, glutathione synthesis is important in preventing oxidative stress [54], and therefore, the downregulation observed here appears counter-intuitive. However, the oxido-reduction response in seaweeds does not rely on glutathione as much as it does in terrestrial plants, and seaweeds are able to detoxify cells via the excretion of ROS or the use of other enzymes to reduce ROS into oxygen and water [69]. It is therefore possible that *F. vesiculosus* relies more on other detoxification mechanisms than on glutathione.

## Conclusions

Our study supports the existence of geographic variation in tolerance to hyposalinity in *F. vesiculosus*. We showed that physiological acclimation to the projected hyposalinity differed strikingly between algal populations, which suggests the presence of genetic variation among populations in the regulation of gene expression. This highlights the importance of considering intraspecific genetic diversity and variance in tolerance to environmental changes when predicting organismal responses to climate change. In the population that showed a substantial acclimation response, we observed that acclimation to hyposalinity involved major adjustments to most of the main metabolic activities. Among these were inhibition of photosynthetic activity, increased metabolism, changes in the membrane composition and structure, and a pronounced anti-oxidative response. Taken together, these results reveal for the first time the genetic mechanisms behind the regulation of osmotic activity, and provide evidence for selection for and possibly local adaptation of genes coding for salinity acclimation responses in this species.

## Methods

### Sample collection

We collected *F. vesiculosus* from Parainen (N 60°13′10.5″, E 22°05′52.3″) on the 11th of May 2016 and from Rauma (N 61°0.5′17.5″, E 21°18′11.1″) on the 10th of May 2016 from a depth of 0.5–1.5 m. No permission for the collection was needed according to the Finnish national guidelines. From each population, we sampled ten individuals, with at least 10 m between samples. Herein, one individual is defined as all apical tips of a thallus growing from a single stem attached to a holdfast. We measured ambient salinity and temperature in both locations (Parainen: 5 PSU, 12.5 °C; Rauma: 5.3 PSU, 13 °C). We stored algae in coolers between wet paper tissues for transportation to the University of Turku. There, we carefully rinsed the algae with freshwater to remove associated grazers and epiphytes and we maintained them in their native salinity and temperature until the experiment started. Samples were left unsterilized to avoid removal of the micro-epibionts such as associated bacteria, as this could influence the algal physiology, thus contributing to the algal acclimation capability [4, 70, 71].

### Conditions for gene expression and sample preparation

We examined the effect of hyposalinity on gene expression by exposing algae to current (5 PSU) and predicted future salinity (2.5 PSU) conditions in an indoor aquarium experiment. The current condition reflected the salinity and temperature conditions recorded during the sampling. The condition used for the hyposalinity stress was obtained from the model RCAO-ECHAM-A2-REF developed in Meier and Eilola [12]. According to this model, the average salinity of the coastal areas around our sampling sites will drop in the upcoming future (2070–2099). We used two separate aquarium racks to expose the algae to the two different salinities. Each aquarium rack consisted of a bottom tank (~ 300 L) and three 24-L aquaria. Seawater was pumped from the bottom tank to the aquaria, from where it flowed back into the bottom tank. Seawater was cleaned first by an acrylic filtration unit (SCHURAN Jetskim 120) that was equipped with a mechanical and biological filter, then by a protein skimmer, and finally by UV radiation. Each bottom tank was equipped with a chiller/heater to regulate the water temperature (10 °C for both aquarium racks). We obtained the salinity for future climate conditions by diluting seawater with distilled water. To ensure ample nutrient availability, we added an enriched seawater medium, composed of micro- (trace metals and vitamins) and macro-nutrients (phosphate and nitrogen), to the bottom reservoirs [72]. Macronutrients were added to mimic the in situ surface concentrations present in the Archipelago Sea from September to April (SYKE, Finnish Environment Institute).

Individual thalli were split into two similar-sized ramets, one of which was randomly distributed in an aquarium in current salinity conditions and the other in an aquarium in future salinity conditions. In order to prevent the ramets from floating, a small ceramic weight was attached to each. Initially both racks were established at 5 PSU (current salinity). In the future-salinity aquaria, we decreased the salinity to 2.5 PSU slowly over the course of 3 days, then maintained this new salinity for 24 h. We then sampled thalli from both salinities by cutting the apical tips, wrapping them individually with aluminium foil, and flash-freezing them in liquid nitrogen. By lowering the salinity slowly over 3 days and keeping the algae in 2.5 PSU for 24 h, we ensured that we were not measuring immediate stress effects, but rather, changes in gene expression that took place during the acclimation process. Samples were subsequently stored at − 80 °C until RNA extraction.

### RNA extraction, sequencing, and pre-processing

Total RNA was extracted using a modified protocol from Pearson et al. [73]; we started with freeze-dried tissue and added an initial acetone wash step as in Panova et al. [74]. We treated the RNA with RNAse-free DNAse-I according to the manufacturer’s instructions (Qiagen) to remove any contaminating DNA. The concentration and quality of RNA was assessed using the 2100 Bioanalyser (Agilent). Of the 20 samples, 14 passed the RNA quality-control threshold, three of them from Rauma population and four from Parainen, each of them replicated in both conditions (Table 1). These samples were sent to the National Genomics Infrastructure (NGI) facility in Stockholm for library preparation and sequencing (Table 1). The libraries were generated with the Illumina TruSeq Stranded mRNA sample preparation protocol with poly-A selection and average insert sizes of 369–476 bp. The indexed libraries were pooled in equimolar amounts and sequenced in one lane of an Illumina HiSeq 2500 apparatus in High Output V4, PE 2 × 125 bp mode. The sequencing generated between 11.98 and 24.16 million raw reads per sample (Table 1). Raw reads from this study are deposited in the NCBI SRA database under accession numbers SRP144722.

Quality control of the raw reads was performed using FASTQC v 0.11.5 software (www.bioinformatics.babraham.ac.uk/projects/fastqc). We quality-filtered the reads in all the samples with Trimmomatic software v. 0.32 [75]. First, bases from the beginning and the end of each read that fell below a quality score of three were trimmed. Second, we used a sliding-window approach with a window size of four bp to remove bases with an average quality score below 15. Adapter sequences were removed using Cutadapt v. 1.9.1 [76] software, and reads shorter than 50 nt were discarded. After trimming, the reads were again visualised using FASTQC.

### De novo transcriptome assembly and annotation

Using all cleaned reads, we produced a de novo transcriptome assembly with the Trinity assembler v. 2.3.2 [77]. We applied in silico read normalisation with maximum coverage set to 30. In assembly, k-mer size was set to 30 and the remaining Trinity parameters were kept as default. Assembly statistics were calculated using the TrinityStats.pl script and the completeness of the assembly was assessed with BUSCO (Benchmarking Universal Single-Copy Orthologs) v. 1.22 [78] against the “eukaryota_odb9” reference set. TransRate v. 1.0.1 [79] was used to evaluate the assembly and remove transcripts that were not supported by read mapping. Transcripts identified as “good” by TransRate were further analysed with TransDecoder v. 2.0.1 [80] to predict the likely coding regions. Assembly statistics and completeness of the filtered assembly were calculated as above and compared to the original assembly. We also compared this assembly to a reference set of 24,486 proteins of the brown algae *Ectocarpus siliculosus*, the genome of which has been sequenced (http://bioinformatics.psb.ugent.be/orcae/overview/EctsiV2), using Conditional Reciprocal Best Blast algorithm within TransRate [79].

For annotation we retained one isoform per gene, the one with the highest read support as identified with the Trinity utility “filter_low_expr_transcripts.pl” and option --highest_iso_only. Annotation of this final transcriptome assembly were performed within the Blast2GO pipeline [81] and included Blastx comparison of sequences to the NCBI nr protein database, GO (Gene Ontology) mapping, InterProScan search and merging of BLAST and InterProScan annotations, applying the default parameters in Blast2GO. Finally, the transcripts were mapped to KEGG pathways (http://www.genome.jp/kegg/pathway.html).

### Genotyping of individuals

*Fucus vesiculosus* can reproduce asexually and clonal individuals may constitute a large proportion in some Baltic populations [82, 83]. The subject populations of this study have also been found harbouring clonality [84] and to ensure that the individuals in our experiment represent different genotypes, we performed transcriptome-wide genotyping of SNPs (Single Nucleotide Polymorphisms). Cleaned reads were mapped to the transcriptome assembly using bowtie2 with the default settings [85] and bam files for the same individual from the future and present conditions were merged. From bam files, a bcf file was generated with samtools mpileup [86], discarding positions with base quality and/or mapping quality < 20. The genotypes were called with bcftools call –c option (https://samtools.github.io/bcftools/bcftools.html) and filtered for min read depth = 8 with vcfutils.pl varFilter. Non-biallelic SNPs were discarded and Principal Component Analysis (PCA) based on SNP allele frequencies was done using R package “adegenet” [87]. Finally, clustering of the individuals was done using Nei’s genetic distance and Neighbor-Joining algorithm in R package “poppr” [88, 89]. Differences in the hyposalinity response between the two populations can result from different genetic background, including allelic variation in the important genes. While the limited number of individuals per population used in the experiment precluded a genome scan, we tested the hypothesis that genes, involved in hyposalinity response show higher genetic divergence between the two studied populations than other genes by comparing the number of SNPs that appear to be fixed for different alleles in the two populations. Bam files, described above, were filtered for min depth = 8 using vcfutils.pl and non-reference SNPs were identified with --non-ref-af option in vcftools [90], applying minimum frequency of non-reference alleles = 0.99. Subsequently, we calculated number of fixed SNPs for each gene, and compared average values between differentially regulated and non-regulated genes. We used a G^2^ test to check whether the amount of DE and non-DE genes was dependent on the amount of fixed SNPs separately for each population using the R package DescTools [91].

### Gene expression analysis

We estimated the transcript abundances based on the pseudo-alignment method *kallisto* [92] implemented within the Trinity utilities “align_and_estimate_abundance.pl” followed by “abundance_estimates_to_matrix.pl”. These data were used to generate a matrix of gene expression values TPM (transcripts per million), normalised across the samples, which was then used for downstream analyses. We identified groups of expression profiles using principal components based on the biological coefficient of variation (read count) between library pairs. This was performed with the R/Bioconductor package “DESeq 2” [93]; the R package “ggplot2” v. 2.2.1 was used to plot the results of the principal components (PCA).

Since the clustering of samples on the plot suggested differences in response between the two populations, we identified the genes with the largest changes in expression. Starting from raw counts, we estimated the library sizes and converted raw counts into expression magnitude (variance/raw means of read counts^2^). Then, we determined the genes that varied in read count from the 95% IC, which highlighted 799 significant outliers.

We estimated the amount of variance in gene expression among populations by ANOSIM (analysis of similarity), which tested distances between populations, salinity conditions, and the interaction population **×** salinity) implemented in the R package “vegan” v. 2.0.3 [94]. This was performed with 999 permutations based on the 799 most-variable genes (according to normalised fold changes). This test showed a significant population **×** salinity interaction (see Results); consequently, we performed the analysis of differentially expressed genes with “DESeq 2” separately for each population. We used a generalised linear model (GLM) with a negative binomial distribution to test the difference in gene expression among salinity treatments for each gene using Wald statistics. We set salinity as a fixed factor (two levels: present and future), nested within individual, and read counts for each gene as the response variable. We corrected the results for multiple testing using the Benjamini-Hochberg procedure, set the false discovery rate (FDR) at a significance threshold of α < 0.05, and applied an absolute log_2_ fold change (FC) cut-off of > 1 (corresponding to expression changes greater than two-fold).

### Comparison with genes involved in hyposalinity response in other brown algae

To date, two studies have looked at the gene expression response to low salinity in two species of brown algae: *Ectocarpus siliculosus* [32] and *Sargassum fusiforme* [47]. Differentially regulated genes reported in these studies were compared to differentially expressed (DE) genes in *Fucus*. We retrieved corresponding protein and/or unigenes sequences from https://bioinformatics.psb.ugent.be/gdb/ectocarpus/Archive/ for *Ectocarpus* and from NCBI for *Sargassum*, and compared them to *Fucus* DE genes by tblastn and tblastx with e-value =1e-3.

## Supplementary information

**Additional file 1.** list of genes differentially expressed due to hyposalinity for Parainen population (FDR < 0.05 and |log_2_FC| > 1).

**Additional file 2.** list of genes differentially expressed due to hyposalinity for Rauma population (FDR < 0.05 and |log_2_FC| > 1).

**Additional file 3 **list of DE genes found by the present research and previously described in *Ectocarpus* and *Sargassum* in response to hyposalinity.

**Additional file 4.** PCAs obtained by plotting the allele frequencies in all SNPs plotting the axes a) 1 and 2, b) 3 and 4, c) 1 and 5.

**Additional file 5 **Neighbor Joining tree for *Fucus* populations studied in the present research. The standard genetic distance of Nei [95] was used.)

## Data Availability

Scripts and information for setting up the analysis can be obtained from the authors upon request. The raw data supporting the conclusions of this article are available in the NCBI SRA database under accession numbers SRP144722.

## Abbreviations

ANOSIM: Analysis of similarity
BUSCO: Benchmarking Universal Single-Copy Orthologs
FDR: False discovery rate
GLM: Generalised linear model
GO: Gene ontology
NGI: National Genomics Infrastructure
PSU: Practical salinity units
RNA-seq: RNA-sequencing
SNP: Single nucleotide polymorphism
TPM: Transcripts per million
