# Supplementary figures and images for: Gene regulatory response to hyposalinity in the brown seaweed Fucus vesiculosus
Source: BMC Genomics. 2020 Jan 13;21:42. doi: 10.1186/s12864-020-6470-y (PMC6958763; doi:10.1186/s12864-020-6470-y)

Additional file 4


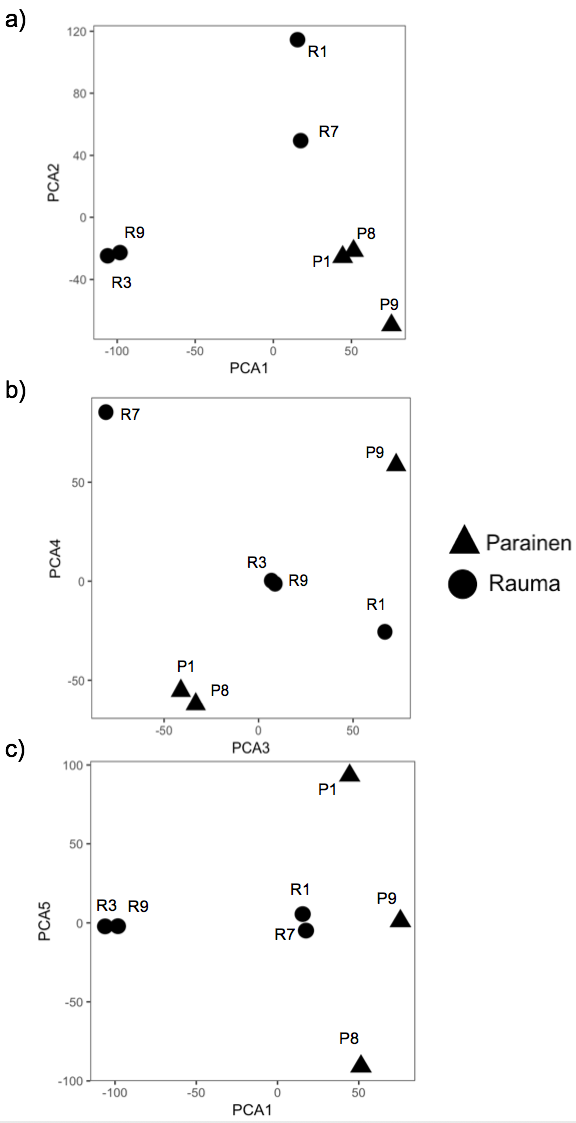

Supplement: Supplementary file 4 — Additional file 4. PCAs obtained by plotting the allele frequencies in all SNPs plotting the axes a) 1 and 2, b) 3 and 4, c) 1 and 5. [file 12864_2020_6470_MOESM4_ESM.docx]

Additional file 5


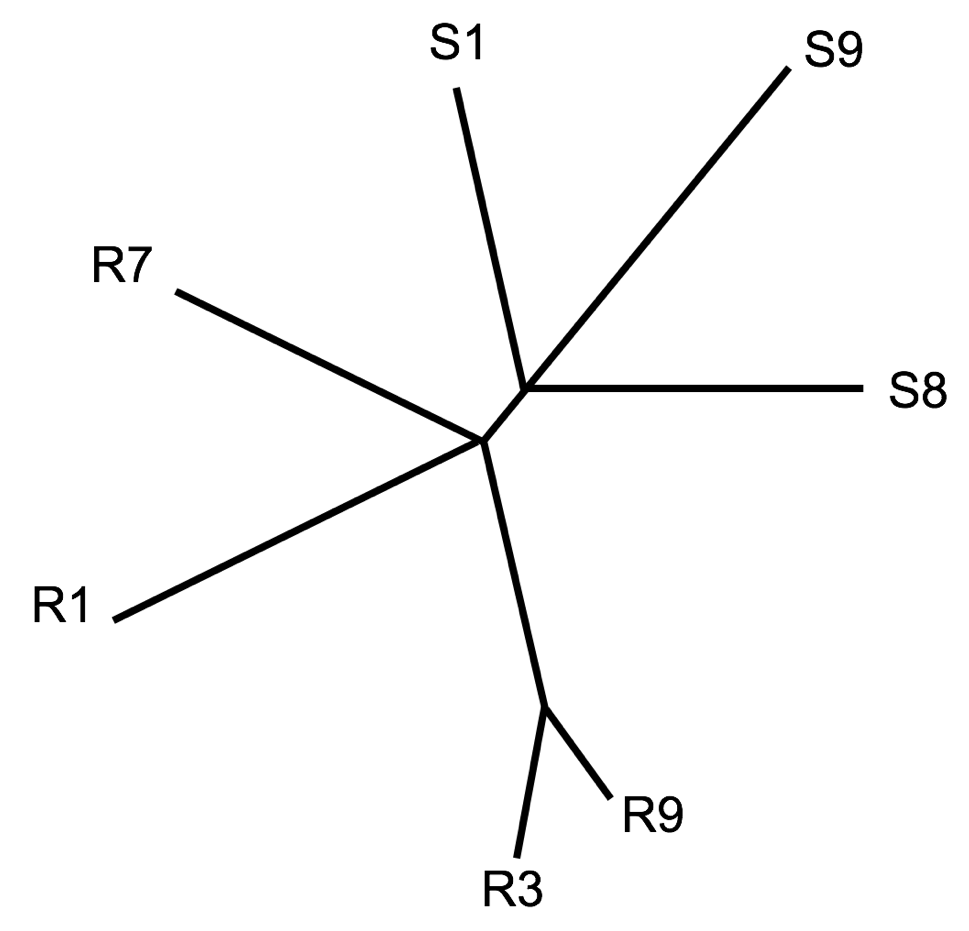

Supplement: Supplementary file 5 — Additional file 5 Neighbor Joining tree for Fucus populations studied in the present research. The standard genetic distance of Nei [95] was used.) [file 12864_2020_6470_MOESM5_ESM.docx]
